# Editorial: COVID-19 and diabetes, volume II

**DOI:** 10.3389/fendo.2023.1136997

**Published:** 2023-01-16

**Authors:** Hamad Ali, Mohamed Abu-Farha, Susanna Hofmann

**Affiliations:** ^1^ Health Sciences Center, Kuwait University, Jabriya, Kuwait; ^2^ Dasman Diabetes Institute, Kuwait City, Kuwait; ^3^ Helmholtz Center München, Helmholtz Association of German Research Centres (HZ), Munich, Germany

**Keywords:** COVID-19, diabetes, SARS – CoV – 2, prognosis, ketoacidosis

People with type 1 or type 2 diabetes mellitus have been highly affected by COVID-19 as they are at a higher risk of developing COVID-19-related complications. The relationship between diabetes mellitus and COVID-19 is complex and multidirectional, nonetheless our efforts to better understand its pathophysiology is evolving at a rapid pace. Individuals with diabetes mellitus are known to be more susceptible to contracting severe COVID-19 and SARS-CoV-2 infection. However, there is also evidence that COVID-19 may influence the pathophysiology of diabetes, affecting blood glucose control not only in those already pre-disposed or living with diabetes but also in those without the disease.

Diabetes mellitus is one of the main comorbidities contributing to a worse COVID-19 prognosis and outcome. Stidsen et al. showed that the risk of hospitalization due to COVID-19 was higher in patients with diabetes compared to non-diabetics during pandemic waves in Denmark. Understanding the pathophysiological relationship between severe COVID-19 outcome and diabetes remains to be a progressive area of research. A review by Landstra et al. suggested that the poor prognosis of COVID-19 in diabetic patients can be linked to the comorbidities and risk factors normally present in diabetic patients. Al-Sayyar et al. adapted a comparative approach between different respiratory tract infection in people with diabetes including Tuberculosis and Influenza to understand the susceptibility of severe COVID-19 in people with diabetes. They reviewed and proposed that several mechanisms which might be responsible for increased susceptibility of severe COVID-19 outcomes in people with diabetes are shared with previously described respiratory infections including Influenza A virus and Mycobacterium tuberculosis.

Xie et al. reviewed current understanding of the complex relationship between diabetes and COVID-19. They provided an update on the two-way interactions between diabetes and COVID-19 and treatment strategies for COVID-19 severe cases from the perspective of ACE2. This bidirectional relationship was investigated by Pelle et al. where they showed that the expression of ACE2 receptors on pancreatic beta cells allow the virus to target the pancreas directly which might lead to hyperglycemia in patients with and without diabetes. This topic was also studied in a review by Shao et al. where, in additional to the direct targeting of the pancreas by the virus, the presence of an abnormal immune response, including an inflammatory storm and imbalances in T cells, could be the cause. Computational and machine learning approaches were utilized to gain insight into the complex relationship between diabetes and severe COVID-19 outcomes. Bassani et al. performed *in silico* evaluation of the possible impact Omicron variant of SARS-CoV-2 in people with diabetes. The adapted computational approach suggested a different clinical relevance of the Omicron variant in people with diabetes. They hypothesized that the affinity between the viral Spike protein and the human receptor ACE2 is higher for Omicron variant when compared to other circulating variants, at the time of the study, in both normal and hyperglycemic environments. A multicenter study in Italy and Spain by Mayneris-Perxachs et al. utilized logistic regression analyses and machine learning algorithms to analyze confounding factors influencing the prognostic value of hyperglycemia related to severe COVID-19 outcomes and found that blood hemoglobin substantially modulates the well-established influence of hyperglycemia on severe COVID-19 outcomes.

Much of the efforts were directed toward better understanding of the impact of antidiabetic agents on COVID-19 patients with diabetes. Metformin is the first-line medication for the management of diabetes. Miao et al. performed a multiracial, multiethnic, urban observational study and found no significance difference in-hospital mortality or length of stay due to COVID-19 in patients group on Metformin. On the other hand, Wong et al. showed that Metformin use was associated with lower mortality rates and lower incidence of hyper-inflammatory syndrome among COVID-19 patients with diabetes. This result agrees with a Meta-Analysis by Kan et al. showed possible association between Metformin use and reduced mortality in COVID-19 patients with diabetes. In a commentary on the previous study, Zhao et al. showed significant association of SGLT2i, DPP4i and GLP1RA use with reduction in COVID-19 mortality risk in people with diabetes. A Bayesian Network Meta-Analysis by Chen et al. assessed the impact of different antidiabetic agents on the infection outcome among COVID-19 patients with diabetes. They found that antidiabetic drugs including Metformin, DPP4i, SGLT2i, and GLP1RA were associated with reduced COVID-19 related mortality. However, the use of insulin was associated with unfavorable outcome. This was also implicated in a systematic review and meta-analysis by Yang et al. where a meta-analysis on 18 studies revealed that Insulin treatment may be associated with increased adverse outcomes in Patients with COVID-19 and diabetes. In a randomized clinical trial of hospitalized COVID-19 patients with diabetes from Palestine, Abuhasira et al. showed that the use of linagliptin did not show any clinical improvement when compared to patients receiving standard care. Additional research on a larger scale is necessary to verify and examine the specific mechanism underlining the effects of antidiabetic drugs in greater detail in order to improve the effectiveness of care given to COVID-19 patients with diabetes. It is important for doctors to consider the risks and benefits of different medications for diabetes, and to tailor treatment plans to the individual needs of each patient, taking into account the risk of hypoglycemia, acidosis, and gastrointestinal symptoms. The goal is to achieve optimal glycemic control in patients with SARS-CoV-2 infection and improve the outcome.

One of the explored strategies to reduce the risk of severe COVID-19 outcomes in people with diabetes was early identification and strong glycemic control. Early identification of people at risk of COVID-19 severe outcomes is very important to develop effective therapeutic approaches. Moin et al. performed detailed proteomic analysis on patients with differing severity of COVID-19 and identified potential coagulopathy proteins that may predict COVID-19 severe outcomes in people with diabetes. Bajpeyi et al. highlighted that unmanaged diabetes and blood glucose in patients encountering COVID-19 increased the risk of disease severity and prolonged hospitalization. This agrees with a study by Wang et al. where they showed that COVID-19 patients with unmanaged glucose levels, due to undiagnosed diabetes or new onset diabetes due to COVID-19 infection, increased the risk of severe outcomes when compared to people with diabetes and managed glucose levels. A study by Llanera et al. showed that CRP and age are useful predictors on severe outcomes in COVID-19 patients with diabetes. Wang et al. explored the use of other markers not linked directly to diabetes and showed that patients with severe COVID-19 outcomes had lower levels of HDL cholesterol and higher NHR at the time of admission to the hospital. These factors were also found to be associated with severe outcomes in COVID-19 patients with diabetes. Moin et al. hypothesized that levels of proteins related to platelets may be affected in people with type 2 diabetes, potentially making them more prone to thromboembolic responses to infections like COVID-19. In their case control study, they showed that people with T2D were at a higher risk for thromboembolic events due to platelet hyperactivation. If they were also infected with COVID-19, this risk may be further increased, and they may be more likely to have a severe course of the disease. In another study by same group, they showed that strict control of fasting blood glucose might lead to hypoglycemia which in turn increases the levels of sNRP1 and its ligands (VEGF and SEMA3A) and put patient with T2D or obesity at higher risk of COVID-19 infection.

One potential factor that could contribute to the differences in COVID-19 severity among individuals is host genetic background. In a Spanish pilot study, Iñiguez et al. examined whether variations in the angiotensin converting enzyme (ACE) gene, which plays a crucial role in regulating the renin-aldosterone-angiotensin system (RAAS), could be associated with COVID-19 severity. Their genetic analysis of 128 COVID-19 cases reveled hat having the *ACE2* variants rs4341 and rs4343 may increase the risk of a worse outcome from COVID-19. These *ACE2* genetic variations, may be useful indicators of the severity of COVID-19 in patients with underlying conditions such as hypertension, dyslipidemia, or diabetes. Understanding this genetic information could help in the more precise treatment of hospitalized patients infected with SARS-CoV-2.

COVID-19 has been linked to the development of autoimmune conditions. Multiple previous reports have highlighted increased prevalence of new onset of type 1 diabetes during the COVID-19 pandemic but the exact pathophysiological pathways is not entirely understood. Mishra et al. presented a case where COVID-19 is identified as a potential trigger for diabetic ketoacidosis and newly detected pancreatic autoantibodies. Cases of ketoacidosis were also identified following COVID-19 vaccination in patients with T1D. Yakou et al. presented case series of severe ketoacidosis after COVID-19 vaccination in a type 1 diabetes patients on insulin and SGLT-2 inhibitor treatments. They proposed that T1D patients who are receiving intensive insulin therapy and treatment with sodium-glucose transporter medications are at a high risk for developing ketoacidosis, hence, careful consideration should be given to administering the vaccine to this group of patients.

The COVID-19 pandemic and the subsequent lockdowns have had a significant impact on the lifestyles of people all over the world, particularly on those with chronic conditions such as type 1 diabetes (T1D) and type 2 diabetes (T2D). In a study from Saudi Arabia, Al-Daghri et al. showed that lockdowns implemented as a response to the COVID-19 pandemic had a negative impact on the lifestyle and daily routines of individuals with diabetes in Saudi Arabia. The negative impact included psychological problems such as symptoms of depression and physical impact including decreased activity. Thy suggested that novel strategies are needed to support the health and well-being of people with T1D and T2D during future pandemics and lockdowns, and promote healthy lifestyle adaptations in this high-risk group. In another study from Saudi Arabia, Alaqeel et al. conducted a multicenter retrospective cohort study and found that lockdown in Saudi Arabia has had a major effect on children with T1DM and has led to a higher occurrence of DKA, including in children with newly diagnosed T1DM. This may be due to a delay in seeking medical attention. An opinion by Muñoz et al. highlighted that the COVID-19 pandemic has caused chronic uncertainty and psychosocial distress, which can negatively affect the ability of young people with type 2 diabetes (T2D) to manage their condition and access prescribed treatments. They recommended the development of multidisciplinary treatment plans to encounter the psychological burden of the pandemic. On the other hand, Minuto et al. reported that the lockdown in Italy caused a positive and unexpected change in blood sugar control for young patients with type 1 diabetes (T1D). The changes in lifestyle, including a healthier and less stressful environment and the continuation of physical activity, resulted in improved blood sugar control that was proportionate to the patient’s age. These conflicting results could be caused by social or geographic differences among the study participants. These differences could include things like the level of access to healthcare and medical treatment, cultural and societal factors, and environmental conditions.

The COVID-19 pandemic has highlighted the impact of comorbidities like diabetes on the severity of the disease. Diabetes has been shown to lead to worse COVID-19 outcomes and increased mortality. Additionally, COVID-19 infection can lead to new-onset diabetes or worsen glycemic control in people with pre-existing diabetes due to pancreatic damage caused by the virus, the body’s stress response to infection, and the use of diabetogenic drugs like corticosteroids in severe cases. Measures taken to curb the spread of the pandemic, like lockdowns, can also negatively impact individuals with diabetes by limiting access to medical care, exercise opportunities and psychological burden.

## Author contributions

All authors listed have made a substantial, direct, and intellectual contribution to the work and approved it for publication.

